# Can an e-learning course improve nursing care for older people at risk of delirium: a stepped wedge cluster randomised trial

**DOI:** 10.1186/1471-2318-14-69

**Published:** 2014-05-27

**Authors:** Lotte van de Steeg, Roelie IJkema, Maaike Langelaan, Cordula Wagner

**Affiliations:** 1 NIVEL, Netherlands institute for health services research, P.O. Box 1568, Utrecht 3500 BN, The Netherlands; 2 EMGO Institute for Health and Care Research, VU University Medical Centre, Amsterdam, The Netherlands

**Keywords:** Delirium, Education, Nurses, Quality improvement

## Abstract

**Background:**

Delirium occurs frequently in older hospitalised patients and is associated with several adverse outcomes. Ignorance among healthcare professionals and a failure to recognise patients suffering from delirium have been identified as the possible causes of poor care. The objective of the study was to determine whether e-learning can be an effective means of improving implementation of a quality improvement project in delirium care. This project aims primarily at improving the early recognition of older patients who are at risk of delirium.

**Methods:**

In a stepped wedge cluster randomised trial an e-learning course on delirium was introduced, aimed at nursing staff. The trial was conducted on general medical and surgical wards from 18 Dutch hospitals. The primary outcome measure was the delirium risk screening conducted by nursing staff, measured through monthly patient record reviews. Patient records from patients aged 70 and over admitted onto wards participating in the study were used for data collection. Data was also collected on the level of delirium knowledge of these wards’ nursing staff.

**Results:**

Records from 1,862 older patients were included during the control phase and from 1,411 patients during the intervention phase. The e-learning course on delirium had a significant positive effect on the risk screening of older patients by nursing staff (OR 1.8, p-value <0.01), as well as on other aspects of delirium care. The number of patients diagnosed with delirium was reduced from 11.2% in the control phase to 8.7% in the intervention phase (p = 0.04). The e-learning course also showed a significant positive effect on nurses’ knowledge of delirium.

**Conclusions:**

Nurses who undertook a delirium e-learning course showed a greater adherence to the quality improvement project in delirium care. This improved the recognition of patients at risk and demonstrated that e-learning can be a valuable instrument for hospitals when implementing improvements in delirium care.

**Trial registration:**

The Netherlands National Trial Register (NTR). Trial number: NTR2885.

## Background

Delirium is a common complication among older hospitalised patients. Approximately 25% of patients aged 65 and over experience delirium during a hospital stay [1]. The incidence is significantly higher among specific patient groups, such as surgery patients [2]. Delirium in older patients is associated with a longer stay in hospital, functional decline, admission to long-term care, and higher mortality [3-6]. However, studies show that healthcare professionals often fail to recognise delirium during a hospital stay [7,8]. This might be explained by a lack of knowledge of delirium among physicians and nurses [1,9-11].

The Frail Elderly Project (FEP) is part of a national patient safety programme launched in the Netherlands in 2008 [12]. The FEP seeks to improve care for patients aged 70 and over, and includes a delirium care guideline for older hospitalised patients. The project and its guidelines are based on existing evidence regarding care for older patients, as well as expert opinion. The FEP delirium guideline primarily aims to improve early recognition of older patients at risk of delirium through risk screening (Table 1). This gives healthcare professionals the opportunity to take action to identify and minimise risk factors.

**Table 1:** Screening instrument for delirium from the Frail Elderly Project


Risk screening for all patients aged 70 and over.	
Three questions for the patient and/or family or caregivers, asked by nursing staff:	
1.	Do you experience memory problems?
2.	Have you needed help with self care in the last 24 hours?
3.	Have you experienced periods of confusion during earlier hospital stay or illness?
One or more questions answered with ‘yes’ indicates a risk of developing delirium.	
**Possible nursing interventions for at-risk patients**	
1.	Observation with the Delirium Observation Screening scale
2.	Prevent dehydration, infections, electrolyte disturbances et cetera
3.	Adequate treatment of pain
4.	Preserve nutritional level
5.	Inform patients’ family
6.	Improve sensory perception

Previous research has demonstrated the difficulty of putting guidelines into practice [13-15]. Although the FEP provided hospitals with advice on the implementation of the guidelines, there were indicators that implementation was not going smoothly [16]. A lack of knowledge and a failure to recognise patients with delirium have been identified as possible causes of poor care [7,10]. An educational tool such as e-learning could be a valuable tool for improving delirium care. E-learning is increasingly used in health care as a means of educating large groups of professionals [17,18]. A review by Cook et al. [19] has shown that the use of e-learning or ‘internet-based education’ is associated with a positive effect on the knowledge, skills, and behaviour of healthcare professionals, as well as on patient outcomes. Computer-assisted learning aimed specifically at nurses has produced less clear-cut results [18].

The aim of this study was to determine whether e-learning can be an effective means of improving the implementation of a quality improvement project. The primary objective was to investigate whether offering nursing staff an e-learning course in delirium care increased the adherence to the FEP guideline. A further objective of the study was to investigate the impact of the course on nurses’ knowledge of delirium.

## Methods

The rationale and design of this study has been described previously in detail [20].

### Intervention

The intervention we studied was an e-learning course on delirium geared towards hospital nursing staff. This course was developed by a commercial publisher (Noordhoff Publishers), in collaboration with a Dutch hospital [21]. The researchers selected this e-learning course for the study and requested and received permission from the publisher for its use. The content of the e-learning course was consistent with the Dutch guidelines regarding delirium care - including the FEP delirium guideline [12,22]. The course contained information on subjects such as clinical features of delirium, risk factors, diagnostics, prevention and treatment (Table 2). It also incorporated case studies and short tests for self-assessment, to facilitate the learning experience.

**Table 2:** Content of the delirium e-learning course

**Chapter**	**Content**
I. Introduction	i. Introduction on the e-learning course, the patients from the case studies and the subject
II. What is delirium?	i. Introduction on the goals and content of the chapter
	ii. Definition of delirium, its clinical features and course
	iii. Risk patients, predisposing and precipitating risk factors, and prevention
	iv. Consequences of delirium
III. Risk screening	i. Introduction on the goals and content of the chapter
	ii. Predisposing and precipitating risk factors and risk screening
	iii. Recording and discussing delirium risk of a patient
IV. Preventive interventions	i. Introduction on the goals and content of the chapter
	ii. Short overview of preventive medical interventions
	iii. Preventive nursing interventions
V. Early recognition and diagnostics	i. Introduction on the goals and content of the chapter
	ii. The importance of early recognition of delirious patients
	iii. Delirium Observation Screening scale
	iv. Confusion Assessment Method - ICU
	v. Delirium and dementia, delirium tremens and delirium caused by medication
VI. Treatment and care	i. Introduction on the goals and content of the chapter
	ii. Focus of treatment and disciplines involved
	iii. Medical treatment
	iv. Nursing interventions regarding treatment and care
	v. Aftercare
	vi. Delirium in the terminal or palliative phase
VII. More information	i. References to guidelines, rapports and other sources of information on delirium

At the time of this trial, the FEP delirium guideline was being implemented by Dutch hospitals independently of this study. We selected an e-learning course which offered the hospitals participating the opportunity of supporting their own implementation process with a course that educates nurses in delirium care.

The e-learning course had two goals: to create or increase awareness about delirium and the associated risks; and to increase knowledge about delirium care. The nurses on the wards which participated received online access to the course for a period of three months. The estimated time needed to complete the course and the test of knowledge that preceded and followed the course, was four hours.

The e-learning course was introduced to the nursing staff during meetings in each hospital in order to optimise the nurses’ participation. In order to stimulate participation further, e-mail reminders were sent to nurses who had not completed the course within one month and again to nurses who had not completed the course within two months [23]. In addition, each ward was provided with a monthly overview of the use and completion of the e-learning course. All the nurses who completed the knowledge test successfully by following the course and answering 80% of the questions correctly received a certificate. If nurses scored less than 80% on the test following the course, they had one opportunity to re-take the test.

### The study setting and its participants

Nineteen of the 81 hospitals that were invited initially were enrolled in the trial. The hospitals that responded to the invitation within the inclusion period were included, provided they had already started implementing the FEP. One hospital declined to participate after initially being enrolled in the study. The remaining 18 hospitals participating included two university hospitals, five teaching hospitals, and eleven general hospitals, varying in size and geographical location.

Data were gathered from two wards in each hospital, typically a general medical and a surgical ward. The e-learning trial required data from two groups of participants from each hospital: patients aged 70 and over admitted to one of the participating wards; and the nursing staff employed on these wards. Nursing staff could not blinded as to whether they received the intervention. Patients and data collection staff (research nurses) were blinded to the trial condition. However, it is possible that they were informed of the trial condition through verbal comments from nursing staff.

#### The study design and its randomisation

**Figure 1:**
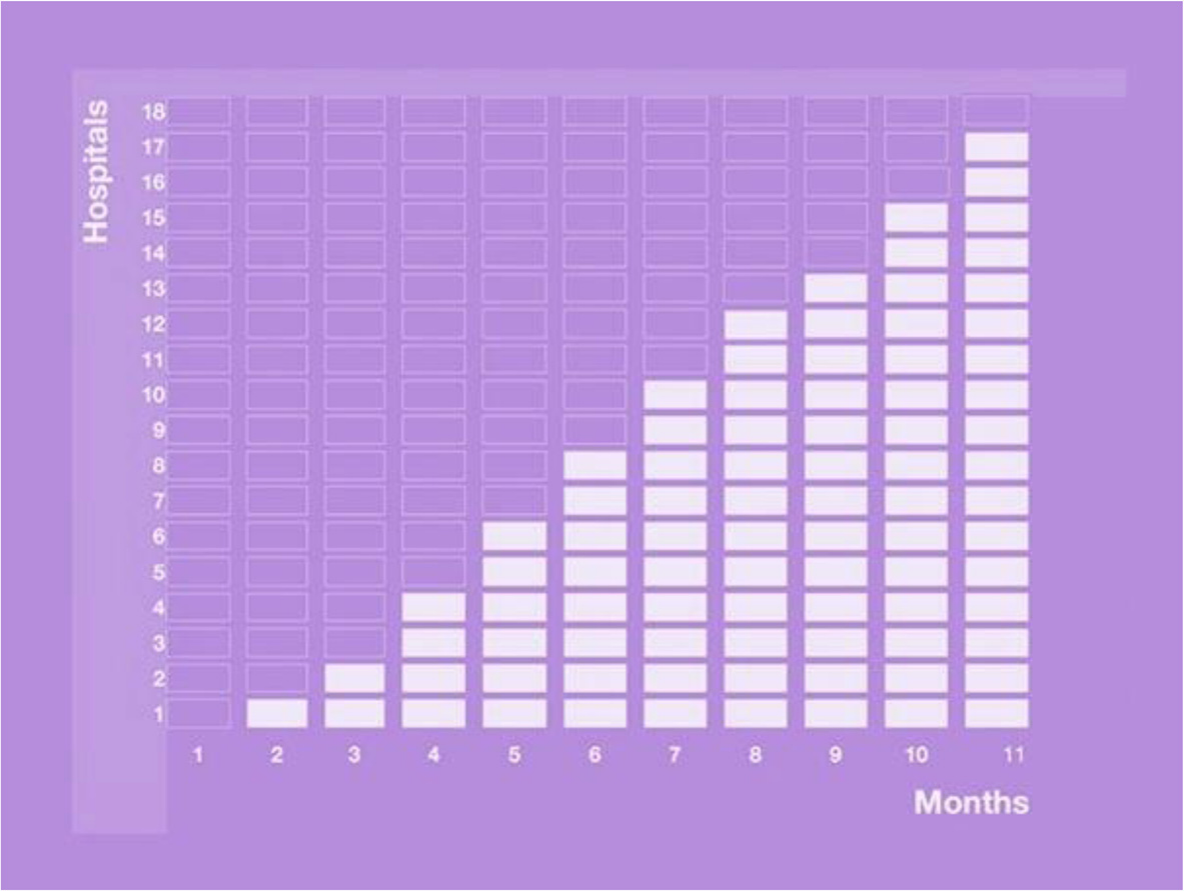
**Diagrammatic illustration of the stepped wedge design.** Each cell represents a moment of data gathering. The empty cells represent data gathering in hospitals without e-learning (control phase). The white cells represent data gathering in hospitals with e-learning (intervention phase) [20].

The study was a stepped wedge cluster randomised trial design [24,25], lasting 11 months. The hospitals that participated crossed over from the control to intervention phase, as illustrated in Figure 1. At the start of the study in May 2011, no hospital had access to the intervention, while at the end in March 2012 all but one hospital had been given access. The order in which the hospitals received the intervention was randomised by assigning computer-generated random numbers to each hospital and subsequently sorting hospitals from the lowest number to the highest. At the start of the study period, all hospitals were informed of the date on which the participating wards would receive access to the e-learning course.

The stepped wedge design resulted in data being gathered from all the hospitals involved, both for the control and the intervention phase. This reduced contamination bias [26].

The initial power calculation for the part of the study focused on the effect of e-learning for nurses on provided care (i.e., screening for delirium risk) resulted in a power of 0.8, based on the following assumptions: 18 participating hospitals (36 wards); an improvement of delirium risk screening of 20% after introduction of the intervention; an alpha of 0.05; a total of 360 patient records for reviewing per month; an intracluster correlation coefficient (ICC) of 0.1.

### Outcomes and data collection

The primary outcome used to determine the effectiveness of e-learning was the percentage of older patients screened for delirium risk. We also looked at the number of nursing interventions received by patients identified as at-risk, as well as the use of the Delirium Observation Screening scale (DOS scale) [27] in this patient group (Table 1). This data was gathered through monthly reviews of patient records, from May 2011 to the end of March 2012. Records were included if the length of stay of the older patient, at the time of data collection, was at least 24 hours. The aim was for research nurses to review ten records per ward per month during the study period. These research nurses did not work for the hospital where they conducted record reviews. Risk screening for delirium was defined as: having used the screening instrument provided by the FEP (Table 1) or an alternative instrument, such as the Groninger Frailty Indicator [28] and the Identification of Seniors At Risk or ISAR [29]. Data on the demographic characteristics of the patients were also gathered.

Secondary outcomes included the percentage of nurses participating in the e-learning course and changes in nurses’ knowledge of delirium after completing the course. These data were gathered using the web-based course itself. Data collection through the e-learning course ended after the final hospitals completed their three month period of access, at the end of June 2012. The characteristics of the nurses working on the wards, such as their age and level of education were collected through the ward managers.

The research nurses conducting the record reviews were blinded for the trial condition of the hospitals. Because the e-learning tool was used by nurses working on the wards participating in the study, the hospitals and wards could not be blinded to the trial condition.

### Statistical analyses

All statistical analyses were performed using STATA 12 and MLwiN 2.25. We compared delirium care in the intervention phase with that of the control phase. We calculated absolute differences and odds ratios using multilevel logistic regression analysis, adjusting for clustering on the ward and hospital level. The outcome were adjusted for hospital type (general or non-general); ward type (general medicine or surgical); patients’ age and sex. Multilevel logistic and linear regression analysis was used to calculate the percentage of nurses participating in the e-learning course, the percentage of participants successfully completing the course, and the changes in the knowledge of delirium, adjusting for hospital type; ward type; and nurses’ age.

If a nurse failed to pass the second test on the first attempt, and made use of the opportunity to re-take the test, results from the second attempt were used in the analysis.

### Ethics

The study had been granted ethical approval by the Vrije Universiteit (VU) University Medical Center in Amsterdam, the Netherlands. According to Dutch legislation, active informed consent was not required.

## Results

### Provided delirium care

During the study period, records from 3,320 patients were reviewed, from all 18 hospitals and 36 wards. Of these, 37 records were excluded from the study because the patient was not admitted, primarily, to one of the general medical or surgical wards participating in the study. A further ten records were excluded because they showed the patient was already suffering from delirium upon arrival at the hospital. Of the 3,273 records included, 1,862 were reviewed during the control phase and 1,411 during the intervention phase (Table 3).

**Table 3:** Patient characteristics and outcome measures, N = 3,273

**Patient characteristics**	**Control phase**	**Intervention phase**	***p***
Included patient records	1,862	1,411	
Patients’ age, mean (SD)	81.0 (6.3)	81.2 (6.5)	0.48
Male patients %	44.4	43.8	0.73
Admitted to a surgical ward %	49.9	47.6	0.20
Admitted to a general hospital %	61.7	59.7	0.27
Delirium risk screening %	50.8	65.4	<0.01
Use of DOS scale %	6.5	10.6	<0.01
Number of nursing interventions	2.1	2.9	<0.01
Recorded delirium diagnoses %	11.2	8.7	0.04

The adjusted delirium risk screening rate was 50.8% (CI 29.9 to 72.4) in the control phase and 65.4% in the intervention phase (CI 60.4 to 70.2) (Table 3). There was a statistically significant effect of the e-learning course for nurses on the risk screening for delirium among older patients, with an OR of 1.8 (CI 1.5 to 2.3, p value <0.01). The intra-class correlation coefficients (ICC) in Table 4 show that 50% or more of the variance in delirium risk screening is due to differences between hospitals.

**Table 4:** Effect of e-learning on the provided delirium care in odds ratios, N = 3,273

**Aspect of care**	**OR**	**CI**	**ICC hospital**	**ICC ward**
Risk screening	1.8	1.5 to 2.3	52.4	1.9
Use of DOS scale	1.7	1.3 to 2.2	24.2	2.1
Recorded delirium diagnosis	0.8	0.6 to 1.0	7.6	0.0

We also found a significant effect on the number of at-risk patients that were observed using the DOS scale, which went up from 6.5% (CI 3.9 to 10.6) in the control phase to 10.6% (CI 8.3 to 13.5) in the intervention phase. The number of nursing interventions that were received by at-risk patients, not including the DOS scale, slightly increased from 2.1 (CI 1.5 to 2.8) in the control phase to 2.9 (CI 2.6 to 3.2) in the intervention phase. While the number of patients for which a diagnosis of delirium was recorded in the patient record saw a decrease, with an OR of 0.8 (CI 0.6 to 1.0, p = 0.04) (Table 4). There were no season fluctuations in diagnosis of delirium (data not shown).

### Participation in e-learning and knowledge of delirium

Of the 18 hospitals participating, one declined the intervention when the trial was already underway, because of organisational circumstances. In total 1,123 invitations for the e-learning course were sent to nurses from 32 wards (17 hospitals); 533 to nurses working in surgical wards and 590 to nurses working in general medical wards (Table 5). The patients from two of the wards participating could be included in the study. However, the nurses could not because we were unable to determine whether they worked on a general medical or surgical ward.

**Table 5:** Nurse characteristics, N = 1,123

**Nurse characteristics**	**Non-participants**	**Participants**	***p***
Included nurses	210	913	
Nurses’ age, mean (SD)*	33.6 (11.9)	35.7 (11.3)	0.04
Male nurses %**	9.6	6.7	0.17
Working in a surgical ward %	56.7	45.4	<0.01
Working in a general hospital %	58.6	60.5	0.61
Level of education: vocational %***	74.1	75.2	0.78
Level of education: university %***	25.9	24.8	0.78

*Missing values: 179.

**Missing values: 69.

***Missing values: 193

Altogether, 90.8% (CI 84.7 to 94.6) of nurses started the course by taking a test of their knowledge of delirium. After attending the course for three months, 92.7% (CI 88.9 to 95.3) of the nurses who had started the course passed the second knowledge test, which signified the successful completion of the e-learning course (Table 6). On average the scores on the second test were 8.9% (CI 8.3 to 9.5, p < 0.01) higher than the scores for the initial test. The corrected average score for the first knowledge test was 79.6% (CI 78.9 to 80.4), compared to 88.6% (CI 88.0 to 89.2) for the second test (Table 6).

**Table 6:** Results of the delirium e-learning course for nurses

	**N**	**%**	**CI**	**ICC hospital**	**ICC ward**
Participation	944	90.8	84.7 to 94.6	8.8	18.7
Successful completion	792	92.7	88.9 to 95.3	10.7	2.0
Mean score initial test	904	79.6	78.9 to 80.4	1.9	0.0
Mean score second test	904	88.6	88.0 to 89.2	1.9	0.0
Mean difference	904	8.9	8.3 to 9.5	1.9	0.0

## Discussion

This stepped wedge trial showed that an e-learning course on delirium did have a significant effect on the nursing staff’s delirium care for older patients, as evidenced by the risk screening. The adjusted screening rate was 50.8% in the control phase, compared with 65.4% in the intervention phase. The e-learning course also showed a significant positive effect on nurses’ knowledge of delirium. An e-learning course on delirium appears to be a valuable addition to the efforts of hospitals to improve delirium care. However, the goal of the FEP, to ensure all older patients were screened for the risk of delirium and all at-risk patients were observed using the DOS scale, was still not achieved.

Our study found a significant increase in the knowledge of delirium after nurses completed the e-learning course. Many experts have emphasised the lack of knowledge regarding delirium in healthcare professionals as a cause of poor delirium care [1,9-11,30]. One study has indicated that nurses do not understand the need for preventive measures because the negative outcomes associated with delirium are not understood well enough [10]. Some experts have suggested that the same is true for clinical and strategic leaders in healthcare, leading to a low priority being awarded to improving the recognition of delirium [30]. Besides the e-learning, and the improvement in knowledge accompanying it, other factors - at the organisational level - might have influenced the delirium care in the hospitals participating. An evaluation of the Dutch patient safety programme found several factors that influenced, positively, the implementation of the patient safety projects. These included, among other things, the presence of an enthusiastic project leader, having the project fit in well with existing guidelines and procedures, and the presence of an electronic patient record [31]. These factors could, and did, differ between the hospitals participating in our trial, and could, potentially, have influenced their adherence to the FEP and the effect of e-learning. This might explain the high ICCs we found.

We did not include a time variable in the model to account for a time effect as suggested by Hussey et al. [25]. The time variable is an important point in stepped wedge designs. One should include the time variable in the model if it can be assumed that delirium is subject to change over time. Balan et al. found a possible seasonal influence on diagnosis of delirium in a geriatric hospital [32]. However, we did not find a seasonal influence on the diagnosis of delirium. This may be due to the relative low amount of events. Besides, several calendar-related effects, change in staff, change in clinical management are hospital dependent and reflected in the clustering on hospital level. Therefore we decided not to include the time variable in the model. Besides, by including several measurement points over time, the timeframe is included in the model itself.

The intervention may be associated with competing events, such as discharge. As the association between delirium and length of hospital stay can go two ways (a longer hospital stay can increase the risk of delirium, while a delirium will likely increase the length of stay) authors felt that such an analysis was beyond the scope of this article. However, it would be an interesting subject for further research in delirium care.

The main strengths of this trial include its large sample size, both of patients and nurses [18], high participation rates for the e-learning course and the inclusion of both surgical and general medical wards. The stepped wedge design enabled a practical evaluation of the effects of an e-learning course, while offering the methodological advantage of using the participating wards as their own control.

The limitations of the study include a potential delay in the intervention effect, resulting from the relatively late uptake of the e-learning course by nurses. All wards were given access to the e-learning course for the period of three months, but most nurses participated in the course only at the end of this period. This would mean that although the wards had entered, officially, the intervention phase of the trial, minimal effects of the intervention could be expected in the first and second month. This could mean that the effect of e-learning on delirium care was actually larger than was calculated. Another limitation is that the first date of delirium diagnosis was not recorded. Therefore it was not possible to study if the intervention reduces the delirium (hazard) rate.

In addition, the nurses were dependent for data collection on information written in the patient record, which might not always have been complete [33,34]. Nonetheless, screening without proper registration would have the same results for the patient as not screening at all because the screening of patients for increased risks or the presence of delirium can only benefit patients when all the relevant care professionals are aware of the outcome. Also, there was no indication that the documentation in patient records differed between the control and intervention phase.

The FEP was part of a national patient safety programme which ended in December 2012, when all hospitals were expected to have implemented, successfully, all aspects of this programme. This external deadline could have had a positive influence on the implementation of the FEP, which could have had an impact on our findings. However, the evaluation of the patient safety programme, has shown that despite this deadline, several of its projects did not show an increase in implementation during 2012 [31]. In addition, any role the external pressure played in hospitals was present both during the control and the intervention phase.

## Conclusion

This study demonstrated that an e-learning course on delirium aimed at nurses from general medical and surgical wards of Dutch hospitals improved the delirium care provided by nurses, and decreased the number of older patients diagnosed with delirium. It showed that by following an e-learning course nurses could build upon their existing knowledge of delirium care. Our findings support the view that educational approaches focussed on increasing awareness of delirium and increasing knowledge on delirium management, are a valuable tool for healthcare organisations in promoting better delirium care for their older patients.

## Abbreviations

FEP: Frail elderly project; DOS scale: Delirium observation screening scale.

## Competing interests

The authors declare that they have no competing interests.

## Authors’ contributions

CW, ML, and LS collectively developed the study design. CW conceived of the study, and led the application for current funding through the Dutch Ministry of Health, Welfare and Sport. LS and RI were involved in the acquisition of data. LS did most of the analyses and wrote the first draft and final revision of this manuscript. CW, ML and RI reviewed the manuscript and provided input into initial and final revisions. All authors read and approved the final manuscript.

## Authors’ information

Lotte van de Steeg and Roelie IJkema are the researchers. Maaike Langelaan is a senior researcher. Cordula Wagner is a professor and program coordinator.

## Pre-publication history

The pre-publication history for this paper can be accessed here:

http://www.biomedcentral.com/1471-2318/14/69/prepub
